# Effects of kiwi fruit (*Actinidia chinensis*) polysaccharides on metabolites and gut microbiota of acrylamide-induced mice

**DOI:** 10.3389/fnut.2023.1080825

**Published:** 2023-02-06

**Authors:** Mengyin Chen, Xuefeng Chen, Ketang Wang, Luyang Cai, Nannan Liu, Duan Zhou, Wei Jia, Pin Gong, Ning Liu, Yujiao Sun

**Affiliations:** ^1^School of Food Science and Engineering, Shaanxi University of Science and Technology, Xi'an, China; ^2^College of Chemistry and Materials Science, Weinan Normal University, Weinan, China

**Keywords:** kiwifruit, polysaccharides, acrylamide toxicity, gut microbiota, metabolomics

## Abstract

**Introduction:**

Kiwifruit (*Actinidia chinensis*) has rich nutritious and medicinal properties. It is widely consumed worldwide for the intervention of metabolism disorders, however, the underlying mechanism remains unclear. Acrylamide, a well-known toxic ingredient, mainly forms in high-temperature processed carbohydrate-rich food and causes disorders of gut microbiota and systemic metabolism.

**Methods:**

This study explored the protective effects and underlying mechanisms of kiwifruit polysaccharides against acrylamide-induced disorders of gut microbiota and systemic metabolism by measuring the changes of gut microbiota and serum metabolites in mice.

**Results:**

The results showed that kiwifruit polysaccharides remarkably alleviated acrylamide-induced toxicity in mice by improving their body features, histopathologic morphology of the liver, and decreased activities of liver function enzymes. Furthermore, the treatment restored the healthy gut microbiota of mice by improving the microbial diversity and abundance of beneficial bacteria such as *Lactobacillus*. Metabolomics analysis revealed the positive effects of kiwifruit polysaccharides mainly occurred through amino and bile acid-related metabolism pathways including nicotinate and nicotinamide metabolism, primary bile acid biosynthesis, and alanine, aspartate and glutamate metabolism. Additionally, correlation analysis indicated that *Lactobacillus* exhibited a highly significant correlation with critical metabolites of bile acid metabolism.

**Discussion:**

Concisely, kiwifruit polysaccharides may protect against acrylamide-induced toxicity by regulating gut microbiota and metabolism.

## 1. Introduction

Acrylamide (AA), a potential human carcinogen, is spontaneously generated during high temperature (>120°C) processing of food (1). French fries, potato chips, and even baked foods such as bread, cakes, biscuits, coffee, and cocoa have been reported to have AA at more than 500 times the maximum allowable limit in drinking water (2). AA, a small organic molecule with strong hydrophilicity, is absorbed by the intestinal tract during food digestion and then transported to various tissues/organs through the blood circulatory system causing damage to the final absorption site (3, 4). AA toxicokinetic studies suggest two major metabolism pathways (5). One is the formation of mercapturic acid adduct from a direct combination of AA and glutathione catalyzed by glutathione S-transferase. Another is the oxidation of AA to epoxidized glycidamide by cytochrome P450 CYP2E1 cyclooxygenase in the liver. Glycidamide can combine with mercapturic acid adduct or hydrolyze to 1,2-dihydroxypropionamide by epoxide hydrolase. AA metabolism is deleterious to the intestinal barrier which causes disorders of gut microbiota affecting host metabolism.

AA toxicity causes oxidative stress, promotes cytokine-induced inflammatory responses, reduces the expression of tight junction proteins, and disrupts the villous structure of the small intestine (6). Furthermore, it can alter the composition of gut microbiota both at the phylum and genus levels, increasing susceptibility to infections, such as *S. typhimurium* infection in mice (7). AA mainly targets the liver, which is the main detoxification organ, and therefore becomes the principal target of AA during its metabolism (8). AA toxicity alters the liver morphology, increases inflammatory cell infiltration, elevates the level of liver function enzymes, and changes the liver and serum metabolites (9, 10).

Thermal processed food habits have increased long-term exposure to low levels of AA, which may cause AA toxicity in consumers (11). Nowadays, natural products are commonly used for overcoming AA toxicity. Crocin and blueberry anthocyanins extracts have been reported to alleviate AA-induced toxicity by reducing oxidative stress (12, 13). Polysaccharides, one of the main active components of natural products, can alleviate AA toxicity. *Ganoderma atrum* polysaccharide was reported to prevent AA-induced hepatic and intestinal damage by reducing oxidative stress and inflammatory response, and maintaining the intestinal barrier and permeability in AA-induced mice (14). Plant polysaccharides, which are high-molecular polymers of monosaccharides linked with glycosidic bonds, are abundant in vegetables and fruits and have various biological activities (15). Usually, plant polysaccharides from natural resources cannot be digested by digestive enzymes and they are digested in the colon, a place where most gut microbiota (16). In the colon, polysaccharides-gut microbiota interactions can regulate the host's metabolism by gut-liver axis, exerting nutritional/pharmacological effects (17). Kiwifruit polysaccharides (KFP) are essential active ingredients of kiwifruits (18). Everyday consumption of kiwifruits, especially the whole fruit with peel, was shown to improve lipid homeostasis and gut microbiota in healthy animals (19). Polysaccharide, as an important component of kiwifruit peel residue, has a variety of biological activities. It is reported that KFP has the strong antioxidant ability. KFP were shown to scavenge DPPH and ABTS free radicals, chelate iron ions, and inhibit lipid peroxidation and protein glycation *in vitro* (20). However, other activities have yet not been tested *in vivo*. Dietary AA is mainly absorbed in the intestine and then enters the liver for detoxification and metabolism (21). The liver and intestine are connected through enterohepatic blood circulation (22). Whether KFP can protect the liver against AA toxicity possibly by regulating the gut microbiota, is largely unclear.

Accordingly, this study was performed in AA-induced mice to evaluate the hepatoprotective effect of KFP and their possible regulatory effect on the host's gut microbiota and metabolism. Furthermore, we analyzed correlations between gut microbiota and metabolites to uncover the underlying hepatoprotective mechanism of KFP. Our results suggest that KFP can be used in functional food as raw material to intervene AA toxicity but also make full use of byproduct of kiwifruit processing can have economic benefits.

## 2. Materials and methods

### 2.1. Chemicals and reagents

Fresh kiwifruits were collected from Shaanxi Province, China. Chemical standards of acrylamide (purity ≥99.0%) was procured from Aladdin Chemical Co. (Shanghai, China). Astragalus polysaccharides were procured from Xi'an Tianxingjian Natural Bio-products Co., Ltd (Xi'an, Shaanxi Province, China). Measurement kits for serum alanine transaminase (ALT), aspartate aminotransferase (AST), alkaline phosphatase (ALP), and triglyceride (TG) were supplied by Nanjing Jiancheng Bioengineering Institute (Nanjing, Jiangsu Province, China). Dibasic sodium phosphate, sodium dihydrogen phosphate, formaldehyde, absolute ethanol, and dimethylbenzene were procured from Sichuan Xilong Scientific Co., Ltd., (Chengdu, Sichuan Province, China). All other chemicals of analytical grade were purchased from Sinopharm Chemical Reagent Co., Ltd., (Shanghai, China).

### 2.2. Preparation of Kiwifruit polysaccharides (KFP)

KFP were isolated from fresh kiwifruits as described previously with slight amendments (23). Briefly, fresh kiwifruits were crushed and the homogenate was filtered through four layers of gauze. The filter residue was collected, dried, and pulverized. KFP from pulverized residue were extracted in water (1:40, m/v). The homogenate was filtrated as before; the filtrate was collected for later use, and the filter residue was subjected to secondary water extraction as before. The two filtrates were combined and then centrifuged. The obtained supernatant was precipitated with 95% ethanol at 4°C overnight. The collected precipitate was deproteinized, purified by an anion-exchange chromatography of DEAE-52, dialyzed, and lyophilized to obtain KFP. The monosaccharide composition and molecular weight (Mw) of KFP were analyzed as reported previously (24). In this study, the average Mw of KFP was 7.60 × 10^5^ Da, and the molar ratio of monosaccharide composition was rhamnose: arabinose: galactose: glucose: galacturonic acid (1:4:11:40:10). Infrared spectroscopy results showed that KFP is a polysaccharide in the form of pyranose and it is connected by a β-glucoside bond.

### 2.3. Animal experiments

**Figure 1:**
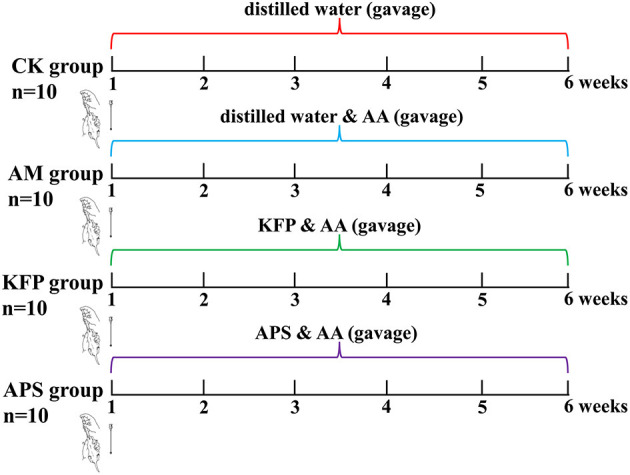
Animal experimental design.

Male, 6-week-old Balb/c mice (20 ± 2 g) were purchased from Xi'an Earnsville Biotechnology (Xi'an, Shaanxi, China) (Production license number: SCXK (jing) 2019-0010). Animals were reared in specific pathogen-free (SPF), standard animal facilities at 22 ± 2°C and 50 ± 10% humidity with caged free rearing, and 12 h light/dark cycle. The mice had *ad libitum* access to a standard diet (AIN-93M) and water. After adapting to the above conditions for 7 days, 60 mice were randomly divided into six groups (*n* = 10 per group): control group (CK), acrylamide model group (AM), low-dose KFP group (KFP-L), medium-dose KFP group (KFP-M), high-dose KFP group (KFP-H), and astragalus polysaccharide positive group (APS). The animal experiment design is depicted in Figure 1. Firstly, mice were intragastrically administered with distilled water (CK group) or 30 mg/kg·bw AA solution (in all other groups). After 1 h, the KFP groups were administrated with 100, 300, and 500 mg/kg·bw KFP solution in KFP-L, KFP-M, and KFP-H groups, respectively; the APS group was administrated with 500 mg/kg·bw APS solution. Meanwhile, CK and AM groups were given distilled water. AA, KFP and APS were administered to mice once a day. We determined the AA dose based on a previous study (25) and preliminary experiments. The entire experiment lasted 6 weeks. During this period, the water intake, food intake, and change in body weight of mice were recorded every week.

After 6 weeks of KFP intervention, all animals were fasted for 12 h and then anesthetized with isoflurane. Mice blood samples were collected and centrifuged at 3,000 rpm for 10 min at 4°C. The obtained serum was stored at −80°C for further analysis. Liver tissue was rapidly removed and weighed. A part of the liver was fixed in 4% paraformaldehyde for tissue sections. The other part was immediately stored at −80°C for further analysis. Also, the cecal contents were collected and stored at −80°C for gut microbiota analysis.

All animal protocols were approved by the Medical Ethics Review Committee of Xi'an Medical College, Xi'an, China. All operations were performed under anesthesia to minimize animal suffering.

### 2.4. Serum biochemical parameters measurement and histopathological examination

The serum levels of ALT, AST, ALP, and TG were measured by commercial assay kits and an automatic biochemical apparatus (BS-460; Shenzhen Mindray Biomedical Electronics) following the manufacturer's instructions. The fresh liver tissues were fixed in 4% paraformaldehyde for more than 24 h, embedded with paraffin, sliced about 4 μm, and finally, stained with hematoxylin and eosin (H&E). The stained liver sections were examined under an optical microscope and the section images were collected by Pannoramic 250 digital slice scanner (3DHISTECH, Hungary). The liver sections were first observed at low magnification for gross lesions and then the selected areas were examined and imaged at 400 x magnification for specific lesions.

### 2.5. Sequencing of cecal microbiota and data analysis

The cecal contents were thawed before DNA extraction using the OMEGA Soil DNA Kit (M5635-02) (Omega Bio-Tek, Norcross, GA, USA). The extracted DNA samples were quantified and subjected to PCR amplification of the bacterial 16S rRNA gene V3-V4 region using the forward primer and reverse primers 338F (5'- ACTCCTACGGGAGGCAGCA-3') and 806R (5'- GGACTACHVGGGTWTCTAAT-3'), respectively. Meanwhile, PCR amplicons were purified with Vazyme VAHTSTM DNA Clean Beads (Vazyme, Nanjing, Jiangsu province, China) and then quantified by Quant-iT PicoGreen dsDNA Assay Kit (Invitrogen, Carlsbad, CA, USA). After the quantification, individual amplicons were pooled in equal amounts and subjected to pair-end 2 × 250 bp sequencing on the Illumina MiSeq platform with MiSeq Reagent Kit V3 at Bioyi Biotechnology Co., Ltd (Wuhan, Hubei province, China). Microbiome bioinformatics was performed with QIIME2 2019.4 with slight modifications with the help of official tutorials (https://docs.qiime2.org/2019.4/tutorials/) (26). Sample alpha diversity was analyzed by QIIME2 and R packages. Alpha diversity include the Chao 1, Shannon and Simpson indices, which represent the richness and diversity of gut microbiota. Beta diversity was analyzed by principal coordinate analysis (PCoA) and nonmetric multidimensional scaling (NMDS) based on the Bray-Curtis metrics. Differential microbiota between groups was identified by linear discriminant analysis (LDA) and LDA effect size (LEfSe) with an LDA threshold of 2. PCoA, NMDS, and LEfSe analysis were evaluated by the R, Ape, Vegan, and Python LEfSe packages.

### 2.6. Serum metabolism analysis

The serum samples were subjected to metabolism analysis as reported previously with minor modifications (27). Briefly, 50 μL serum was extracted with a 200 μL admixture of methanol and acetonitrile to precipitate proteins. The respective admixture containing isotope-labeled internal standard was vigorously vortexed, ultrasonicated, incubated, and then centrifuged. The supernatants were filtered through a 0.22 μm microfiltration membrane and then analyzed by Ultra High-Performance Liquid Chromatography (UHPLC) coupled with Q Exactive Hybrid Quadrupole-Orbitrap mass spectrometer (UHPLC-QE-MS). The quality control sample included an equal amount of all samples. Instrument detection was performed using a UHPLC system (Vanquish, Thermo Fisher Scientific) with a Waters ACQUITY UPLC BEH Amide column (2.1 × 100 mm, 1.7 μm) coupled to Q Exactive HFX mass spectrometer (Orbitrap MS, Thermo). The mobile phase-A consisted of 25 mmol/L ammonium acetate and 25 ammonia hydroxide in water (pH = 9.75); the mobile phase-B was acetonitrile. The Q Exactive HFX mass spectrometer is capable of primary and secondary mass spectrometry data acquisition in information-dependent acquisition (IDA) mode under the control of system software (Xcalibur, Thermo). The ESI source was set to positive and negative modes under the following conditions: capillary temperature, 350 °C; full MS resolution at 60,000; MS/MS resolution as 7,500; collision energy, 10/30/60 in NCE mode; spray voltage, 3.6 kV (positive mode) or −3.2 kV (negative mode).

The raw data were processed by ProteoWizard software and the self-written R package (the kernel: XCMS) for peak identification, peak extraction, peak alignment, and integration. SIMCA (V16.0.2, Sartorius Stedim Data Analytics AB, Umea, Sweden) was used to perform principal component analysis (PCA) and orthogonal projections to latent structures-discriminant analysis (OPLS-DA). The OPLS-DA model was verified by 200 permutation tests; the model quality was assessed by *Q*^2^ and *R*^2^ values. Besides, potential metabolite markers and pathways enrichment analysis were identified from the biochemical databases including HMDB (http://www.hmdb.ca/), PubChem (https://pubchem.ncbi.nlm.nih.gov/), Kyoto Encyclopedia of Genes and Genomes (KEGG; http://www.kegg.com/) and MetaboAnalyst (http://www.metaboanalyst.ca/).

### 2.7. Statistical analysis

Statistical analysis was performed using GraphPad Prism 8 and SPSS 19.0 software (SPSS Inc., Chicago, IL). Differences among the groups were assessed by one-way analysis of variance followed by multiple comparison tests using the least square method or Student's *t*-test. Data are shown as mean ± SD. The correlations between gut microbiome and metabolites were analyzed based on the Spearman rank correlation coefficient. The correlation coefficients were always between −1 and +1. If the absolute value of the correlation coefficient was closer to 1, the linear relationship was better.

## 3. Results

### 3.1. KFP alleviated the negative effect of AA toxicity on body weight and food and water intake in mice

We used KFP intervention at different doses (100, 300, or 500 mg/kg·bw daily for 6 weeks) against AA toxicity in mice. Animals were induced with AA toxicity at 30 mg/kg·bw of AA. The experimental design is shown in Figure 1.

To evaluate the effect of KFP on weight loss of AA-induced mice, the weight of each mouse was measured weekly (Table 1). There was no significant difference in the initial body weight of animals. In the 4th week of the study, the body weight of AM mice decreased significantly compared with other mice. Loss in body weight is a characteristic of AA toxicity, indicating the successful establishment of the AA-mice model. Meanwhile, at 3–6 weeks, the body weight of KFP-M mice was significantly higher than other AA-induced mice (*p* < 0.05), indicating the intervention effect of KFP. The change in the food and water intake of mice during the study are shown in Table 1. Consistent with body weight loss, the food intake of AA-induced mice was lower than CK mice (*p* < 0.05). There was no significant difference in water intake among the animals of the CK, APS, and KFP-M groups. However, compared with KFP-M mice, the water intake of AM and KFP-L mice was significantly higher. Concisely, these results showed that exposure to 30 mg/kg AA negatively affected body weight and food and water intake of mice. However, some of these adverse effects were alleviated by KFP intervention.

**Table 1:** The effects of KFP on body weight, food and water intake in AA-induced mice (mean ± SD, *n* = 10).

	**Body weight (g)**							**Food intake (g per mice per day)**	**Water intake (ml per mice per day)**

**Group**	**0th week**	**1th week**	**2th week**	**3th week**	**4th week**	**5th week**	**6th week**		
	CK	24.51 ± 0.61^a^	25.32 ± 0.89^a^	26.45 ± 1.02^a^	27.91 ± 0.91^a^	28.89 ± 0.95^a^	29.80 ± 1.02^a^	30.13 ± 0.89^a^	4.23 ± 0.10^a^	5.07 ± 0.07^d^
AM	24.12 ± 0.41^a^	24.93 ± 0.36^a^	25.56 ± 0.62^a^	25.81 ± 0.81^b^	23.29 ± 0.95^d^	22.91 ± 0.60^d^	20.85 ± 0.76^d^	3.53 ± 0.05^cd^	5.62 ± 0.03^a^
KFP-L	24.42 ± 0.35^a^	25.46 ± 0.47^a^	26.01 ± 0.61^a^	26.23 ± 0.71^b^	24.78 ± 0.62^c^	25.18 ± 0.59^c^	24.12 ± 0.70^c^	3.56 ± 0.09^cd^	5.54 ± 0.13^ab^
KFP-M	24.19 ± 0.70^a^	25.11 ± 0.66^a^	25.94 ± 0.59^a^	26.21 ± 0.77^b^	25.09 ± 1.06^c^	25.34 ± 1.08^c^	25.33 ± 0.96^c^	3.70 ± 0.08^bc^	5.32 ± 0.03b^cd^
KFP-H	24.31 ± 0.63^a^	25.34 ± 0.89^a^	26.10 ± 0.80^a^	26.16 ± 0.85^b^	25.05 ± 0.97^c^	25.75 ± 0.60^c^	25.53 ± 1.29^c^	3.40 ± 0.04^d^	5.48 ± 0.17^abc^
APS	24.64 ± 0.40^a^	25.50 ± 0.60^a^	26.46 ± 0.78^a^	27.55 ± 0.58^a^	26.76 ± 0.44^b^	27.07 ± 0.63^b^	28.02 ± 1.45^b^	3.84 ± 0.05^b^	5.27 ± 0.06^cd^

Data are expressed as the mean ± SD (*n* = 10). ^a, b, c, d^Mean values in the same column with different letters were significantly different (*P* < 0.05) by a Tukey's test (*n* = 10).

### 3.2. KFP-M intervention produced the best hepatoprotective effect: Serum biochemical and liver histopathology analysis

**Figure 2:**
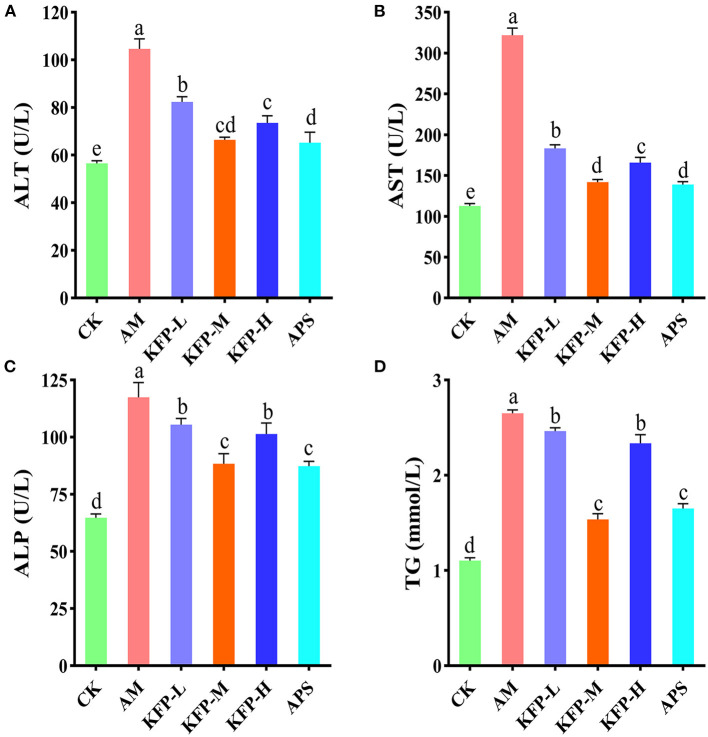
The effects of KFP on serum AST, ALT, ALP and TG in AA-induced mice. **(A)** ALT, **(B)** AST, **(C)** ALP, **(D)** TG. The data are represented as mean ± SD (*n* = 10). Different letters indicate significant differences between groups, whereas the same letters indicate no significant differences. ^a,b,c,d^*p* < 0.05. CK, control group; AM, acrylamide model group; KFP-L, low-dose KFP group; KFP-M, medium-dose KFP group; KFP-H, high-dose KFP group; APS, Astragalus polysaccharide positive group.

Compared with CK mice, the serum levels of AST, ALT, ALP, and TG were significantly higher in AA-induced mice. However, compared with AM mice, these were significantly lower in KFP and APS-treated mice (*p* < 0.05). Notably, KFP-M mice had much-improved biochemical parameters than other groups mice (Figures 2A–D). These results suggested that AA promoted liver damage in AM mice, while different doses of KFP and APS exerted a noticeable protective effect against AA-induced liver damage.

**Figure 3:**
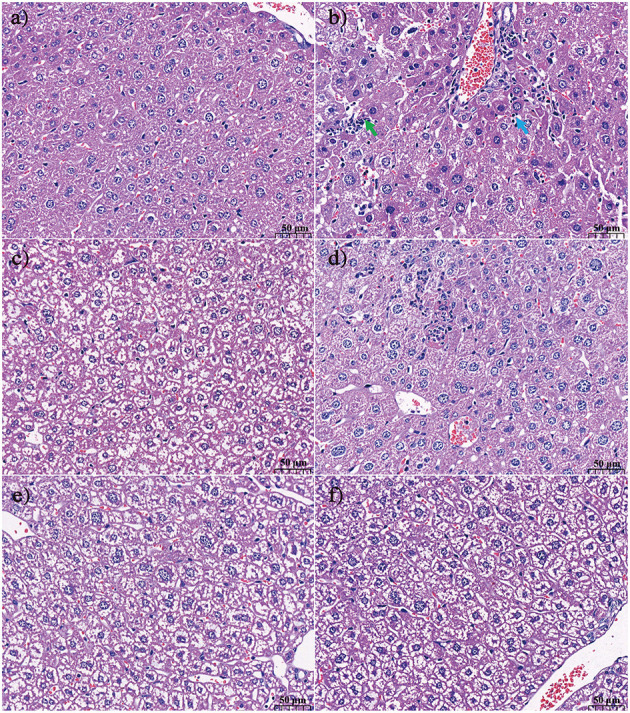
The effect of KFP on the histological examination of liver in the AA induced mice with H&E **(a)** CK, **(b)** AM, **(c)** KFP-L, **(d)** KFP-M, **(e)** KFP-H, **(f)** APS (magnification × 400). Green arrow repersents hepatocyte necrosis and blue arrow represents lymphocytes. CK, control group; AM, acrylamide model group; KFP-L, low-dose KFP group; KFP-M, medium-dose KFP group; KFP-H, high-dose KFP group; APS, Astragalus polysaccharide positive group. Each group consisted of five samples.

The effect of KFP or APS intervention on the liver histology of AA-induced mice is shown in Figure 3. In the CK group, there was no thickening of the liver tissue capsule; the hepatic lobules were not clearly divided; the hepatic cords were arranged neatly; the hepatocytes were arranged radially around the central vein, and the morphology of the hepatocytes was normal. The structure of arteries, veins, and bile ducts was relatively complete (Figure 3a). In contrast, AA treatment resulted in hepatocyte necrosis, increased lymphocyte count, obvious vacuolar degeneration of hepatocytes in the hepatic lobule, lighter staining of degenerated hepatocytes, sparse cytoplasm, and enhanced light transmittance (Figure 3b). KFP-L and KFP-H mice showed slight amelioration of the histopathological effects of AA toxicity, however, degeneration of some hepatocytes was evident (Figures 3c, e). Interestingly, APS and KFP-M mice showed a gradual return to normal histopathological morphology with hepatocyte regeneration (Figures 3d, f), demonstrating the hepatoprotective of KFP at the medium level (300 mg/kg·bw daily for 6 weeks). In all, liver pathological analysis was consistent with serum biochemical analysis.

### 3.3. KFP-M intervention improved gut microbiota health in AA-induced mice

**Figure 4:**
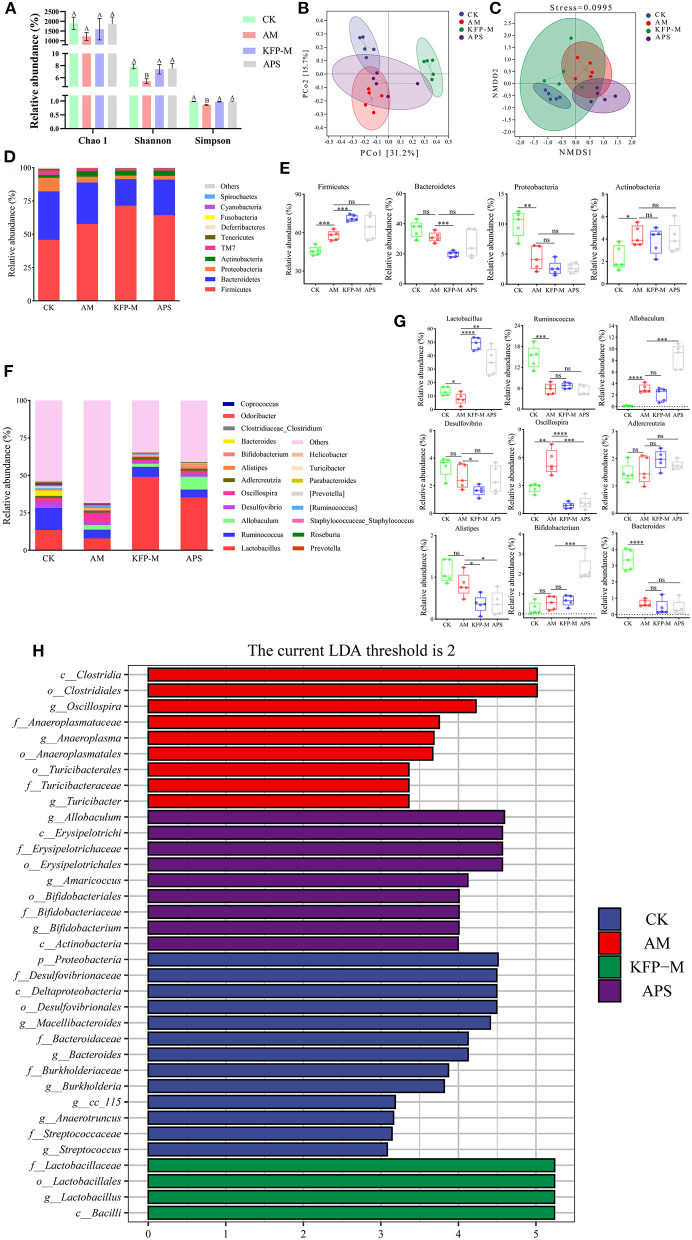
Effect of KFP on gut microbiota composition in AA-induced mice. **(A)** Alpha diversity index for gut microbiota from the CK, AM, KFP-M and APS groups presented by Chao1, Shannon and Simpson indices; Different letters indicate significant differences between groups, whereas the same letters indicate no significant differences. ^A, B^*P* < 0.01 **(B)** Principal coordinate analysis (PCoA) by Bray-Curtis distance. **(C)** Non-metric Multidimensional scaling (NMDS) Bray-Curtis distance (stress = 0.0995). **(D)** The composition of gut microbiota bar plot analysis on phylum levels. **(E)** The relative abundances of Firmicutes, Bacteroidetes, Proteobacteria and Actinobacteria in CK, AM, KFP-M and APS groups were contrasted by Student's *T*-test with significant difference between groups represented as ****p < 0.0001, ***p < 0.001, **p < 0.01, *p < 0.05 and ns represent no significant difference on phylum level. **(F)** The gut microbiota composition bar plot analysis on genus levels. **(G)** The relative abundances of *Lactobacillus, Ruminococcus, Allobaculum, Desulfovibrio, Oscillospira, Adlercreutzia, Alistipes, Bifidobacterium* and *Bacteroides* in CK, AM, KFP-M and APS groups were contrasted by Student's *T*-test with significant difference between groups represented as ***p < 0.001, **p < 0.01, *p < 0.05 and ns represent no significant difference on genus levels; **(H)** The distribution histogram of LEfSe analysis (LDA > 2); The data was expressed as means ± SD (*n* = 5). CK, control group; AM, acrylamide model group; KFP-M, medium-dose KFP group; APS, Astragalus polysaccharide positive group. Each group consisted of five samples.

Next, we examined if KFP hepatoprotective effects were associated with gut microbiota health. The cecal contents of AA-induce mice were analyzed after 6 weeks of KFP-M intervention for microbiota diversity and composition by 16S rRNA gene sequencing. α-diversity analysis was performed based on operational taxonomic units, including Chao1, Shannon, and Simpson indices. As shown in Figure 4A, the Chao 1 index showed no difference among CK, AM, KFP-M, and APS mice, indicating no negative effect of AA toxicity on the richness of mice gut microbiota. Meanwhile, the Shannon and Simpson indices of AM mice were lower than CK, KFP-M, and APS mice, demonstrating that AA toxicity disturbed the diversity of mice gut microbiota. However, KFP or APS intervention significantly improved the diversity of mice gut microbiota. Furthermore, PCoA and NMDS plots were used to evaluate the influence of AA, KFP, and APS on the composition and structure of mice gut microbiota (Figures 4B, C). The results showed a significant deviation between CK and AM mice, indicating that AA altered the composition of mice gut microbiota (Figures 4B, C). The gut microbiota structures of KFP-M and APS mice were entirely different from AM mice, suggesting that KFP-M or APS intervention significantly improved the gut microbiota health of AA-induced mice (Figures 4B, C). To further explore the specific differences, the taxonomic composition of the mice gut microbiome was analyzed at the phylum and genus levels. At the phylum level (Figure 4D), the gut microbiota mainly consisted of Firmicutes, Bacteroidetes, Proteobacteria, and Actinobacteria, accounting for more than 95% of the total bacteria. The Firmicutes were the most abundant bacteria in all mice. Compared with CK mice, in AM mice, the relative abundance of Firmicutes increased from 45.8 to 57.7% (^***^p < 0.001) and Actinobacteria from 2.31 to 4.27% (^*^p < 0.05), while the relative abundance of Proteobacteria decreased from 10.11 to 4.37% (^**^p < 0.01) (Figure 4E). Notably, KFP-M or APS intervention could not reverse these changes. At the genus level, AM and KFP-M mice showed changes in the relative abundance of gut microbiota to varying levels (Figure 4F). In detail, compared with CK mice, AM mice showed prominently increased abundance of *Allobaculum, Oscillospira*, but decreased abundance of *Lactobacillus, Ruminococcus* and *Bacteroides* (Figure 4G; ^****^p < 0.0001, ^***^p < 0.001, ^**^p < 0.01, ^*^p < 0.05). However, KFP-M intervention significantly reduced the abundance of *Oscillospira* and increased the abundance of *Lactobacillus* in AA-induced mice (Figure 4G; ^****^p < 0.0001, ^***^p < 0.001, ^**^p < 0.01, ^*^p < 0.05). Intriguingly, gut microbiota composition did not change at the phylum level but improved at the genus level in KFP-M mice. Concisely, the results suggested that a medium dose of KFP could alleviate gut microbiota dysbiosis in AA-induced mice. LEfSe analysis combined with nonparametric Kruskal-Wallis and Wilcoxon rank sum tests and LDA effect size can help identify the biomarkers. The results of LEfSe analysis are shown in Figure 4H; there were 16, 9, 5, and 11 potential gut microbiota biomarkers (LDA > 2) in CK, AM, KFP-M, and APS mice, respectively.

### 3.4. KFP-M intervention alleviated serum metabolite changes in AA-induced mice

**Figure 5:**
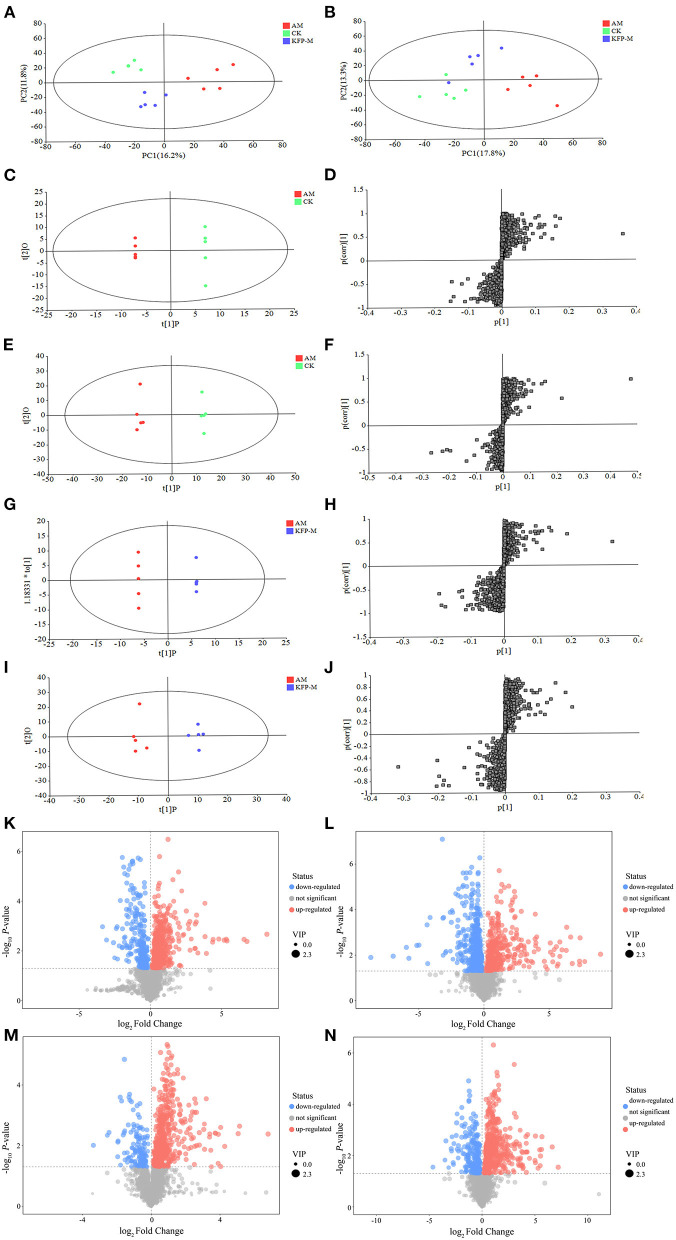
PCA score plots of serum metabolic profiling in positive mode **(A)** and negative mode **(B)**. OPLS-DA score plots of serum metabolic profiling of AM and CK in positive mode **(C)** and negative mode **(E)** and OPLS-DA S-plots in positive mode **(D)** and negative mode **(F)**. OPLS-DA score plots of serum metabolic profiling of AM and KFP-M in positive mode **(G)** and negative mode **(I)** and OPLS-DA S-plots in positive mode **(H)** and negative mode **(J)**. Volcano plots of metabolites between CK vs. AM groups in positive mode **(K)** and negative mode **(L)** and AM vs. KFP-M in positive mode **(M)** and negative mode **(N)**. CK, control group; AM, acrylamide model group; KFP-M, medium-dose KFP group. Each group consisted of five samples.

Considering the remarkable protective effect of medium-dose KFP on weight, serum biochemical parameters (ALT, AST, ALP, and TG), and liver pathology of AA-induced mice, we next examined the metabolites of CK, AM and KFP-M mice by serum untargeted metabolomics. UHPLC-QE-MS was used to find the serum metabolite information in positive and negative ion modes. The metabolite trends across different treatment groups were visualized by PCA. The variance explained by the two principal components (PC) is shown on the X and Y axes in a two-dimensional score plot. In Figure 5A, PC1 and PC2 accounted for 16.2 and 11.8% of the variance in the positive mode. Likewise, in Figure 5B, PC1 and PC2 accounted for 17.8 and 13.3% of the variance in the negative mode. The CK and AM mice groups were clearly separated in PCA score plots, indicating remarkable and massive inter-group variation in serum metabolites. Moreover, PCA analysis showed good separation between the KFP-M and AM groups, indicating a clear difference in their metabolites. The OPLS-DA maximized the differences between groups. In the OPLS-DA model, S-plot can identify the characteristics of inter-group differences. The OPLS-DA score plots manifested clear separations of metabolic profiles between AM and CK groups in both positive and negative modes (Figures 5C, E); the model quality evaluation index under positive ion mode was *R*^2^Y = 0.999, *Q*^2^ = 0.920 (Figures 5C, D), and *R*^2^Y = 0.996 and *Q*^2^ = 0.927 under negative ion mode (Figures 5E, F). The metabolites of KFP-M and AM groups also showed a good separation in both positive and negative ion modes (Figures 5G, I); the model quality evaluation index was *R*^2^Y = 0.999 and *Q*^2^ = 0.780 in the positive ion mode (Figures 5G, H), and *R*^2^Y = 0.974 and *Q*^2^ = 0.785 in the negative ion mode (Figures 5I, J). These values with R^2^Y > *Q*^2^ and *Q*^2^ > 0.5 indicated the stability and reliability of the model.

**Figure 6:**
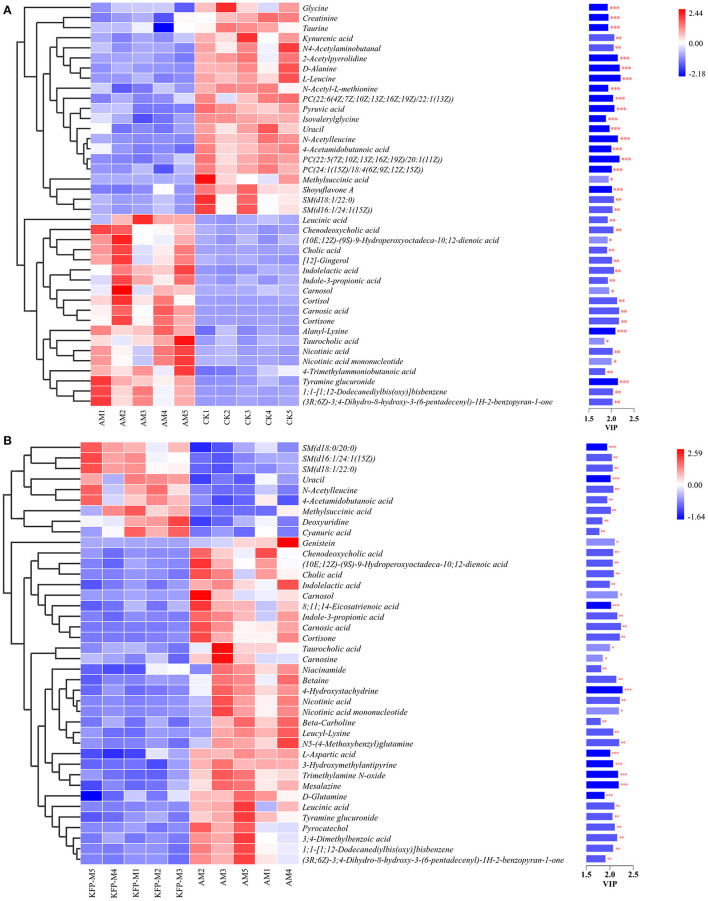
Heatmap of the screened most important 59 metabolites between **(A)** CK vs. AM groups and **(B)** AM vs. KFP-M groups. Significant difference between groups was represented as ***p < 0.001, **p < 0.01, and *p < 0.05. CK, control group; AM, acrylamide model group; KFP-M, medium-dose KFP group. Each group consisted of five samples.

To more intuitively observe the overall distribution of metabolites between CK vs. AM groups and AM vs. KFP-M groups, the visually changed compounds were screened and displayed by volcano plot based on the variable importance in the projection (VIP) values > 1.0, fold change (FC) > 2.0 or < 0.05, and *p*-values < 0.05. The volcano plots showed that compared with CK mice, in AA-induced mice, there were 116 significantly altered metabolites (71 upregulated and 45 downregulated) under the positive mode and 95 significantly altered metabolites (47 upregulated and 48 downregulated) under the negative mode (Figures 5K, L). After the KFP-M intervention, compared with AM mice, in total, 86 (13 upregulated and 73 downregulated) and 77 (17 upregulated and 60 downregulated) differential metabolites were identified under positive and negative modes (Figures 5M, N). To further identify critical metabolites associated with KFP-M intervention, potential metabolic biomarkers were identified through a heatmap combined with VIP scores, which were used to rank the contribution of individual metabolites between different groups. The metabolites with top 40 VIP scores were selected as potential biomarkers, all of them had VIP > 1 and *p* < 0.05. As shown in Figure 6A, these 40 compounds exhibited significant differences between CK and AM groups (*p* < 0.05), 20 of which increased, while the other 20 decreased in AM mice compared with CK mice. Meanwhile, the 40 compounds also exhibited significant differences between AM and KFP-M groups (*p* < 0.05; Figure 6B); 9 metabolites increased and 31 decreased in KFP-M mice compared with AM mice. In addition, among the screened 80 differential metabolites, 21 were common in CK, AM, and KFP-M mice.

**Figure 7:**
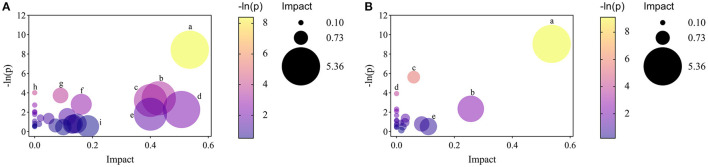
The analyses of related pathways changes by KFP-M. **(A)** AM vs. CK groups. (a) nicotinate and nicotinamide metabolism. (b) taurine and hypotaurine metabolism. (c) pyrimidine metabolism. (d) glycine, serine and threonine metabolism. (e) thiamine metabolism. (f) arginine and proline metabolism. (g) primary bile acid biosynthesis. (h) biosynthesis of unsaturated fatty acids. (i) valine, leucine and isoleucine biosynthesis. **(B)** KFP-M vs. AM groups. (a) nicotinate and nicotinamide metabolism; (b) alanine, aspartate and glutamate metabolism. (c) histidine metabolism; (d) D-arginine and D-ornithine metabolism; (e) D-glutamine and D-glutamate metabolism.

**Figure 8:**
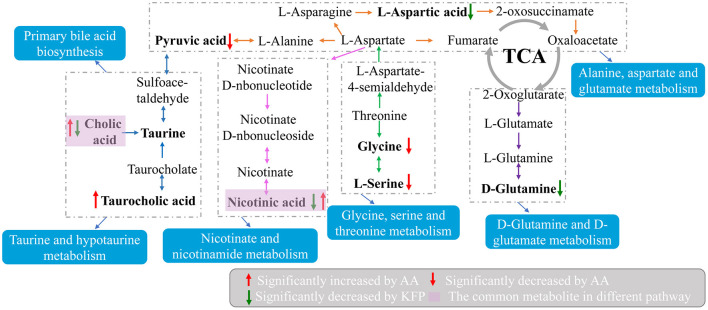
A schematic overview of the connections between effective metabolites and pathways changed by KFP-M.

Moreover, to explore the most correlative pathways and possible biological mechanisms of KFP-M intervention on AA toxicity, the functions of identified metabolites were further analyzed through the KEGG, PubChem, and HMDB databases. As shown in Figure 7A, 9 metabolism pathways were highly enriched in AM mice compared with CK mice, including nicotinate and nicotinamide metabolism, taurine and hypotaurine metabolism, pyrimidine metabolism, glycine, serine, and threonine metabolism, thiamine metabolism, arginine and proline metabolism, primary bile acid biosynthesis, biosynthesis of unsaturated fatty acids, and valine, leucine and isoleucine biosynthesis. Likewise, 5 significantly enriched pathways were observed in KFP-M mice compared with AM mice, including nicotinate and nicotinamide metabolism, alanine, aspartate and glutamate metabolism, histidine metabolism, D-arginine and D-ornithine metabolism, and D-glutamine and D-glutamate metabolism (Figure 7B). Additional details are shown in Supplementary Table S1 indicating 44 different metabolites and 39 metabolism pathways among CK, AM and KFP-M groups. In the nicotinate and nicotinamide metabolism pathway, nicotinic acid mononucleotide, niacinamide, 1-methyl-nicotinamide, and nicotinic acid were significantly increased in AM mice with CK mice. Whereas, the same were significantly decreased after KFP intervention (^***^p < 0.001, ^**^p < 0.01 and ^*^p < 0.05). Meanwhile, nicotinic acid mononucleotide and nicotinic acid were also identified as important metabolites (Figure 6). Compared with the CK group, chenodeoxycholic, allocholic, and cholic acids were increased in AM mice, and while KFP treatment decreased chenodeoxycholic, cholic, and taurocholic acids of the primary bile acid biosynthesis pathway (^***^p < 0.001, ^**^p < 0.01 and ^*^p < 0.05). Meantime, chenodeoxycholic and cholic acids were identified as important metabolites (Figure 6). Compared with CK mice, taurocholic acid of the taurine and hypotaurine metabolism pathway was increased in AM mice and decreased after KFP intervention. In sum, 38 metabolism pathways and 43 differential metabolites were alerted in AM mice compared with CK mice, of which, 21 metabolism pathways and 24 differential metabolites were alleviated by KFP intervention. Therefore, based on the relationships between metabolites and metabolism pathways, a schematic overview of the prominent differential metabolites correlated with enriched metabolism pathways was constructed in Figure 8. These results manifested that KFP-M intervention ameliorated the metabolism disturbances of AA-induced toxicity.

### 3.5. Correlations between gut microbiota composition and serum metabolites

**Figure 9:**
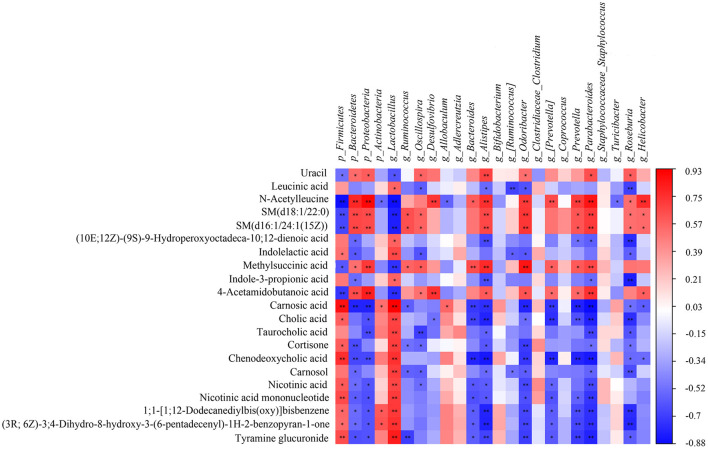
The Spearman correlation analysis of the gut microbiota and serum metabolites. Correlation between the predominant gut microbiota and metabolites at phylum and genus levels among CK, AM and KFP-M groups. The correlation or difference between groups with significance was represented as **p < 0.01 and *p < 0.05. CK, control group; AM, acrylamide model group; KFP-M, medium-dose KFP group. Each group consisted of five samples.

A correlation matrix was established to calculate the Spearman correlation coefficient for the comprehensive analysis of relationships between metabolites and gut microbiota (Figure 9). Twenty four gut microbiota correlated with 21 differential metabolites. For instance, *g_Lactobacillus* was extremely significant correlated with 17 differential metabolites (^**^p < 0.01), including cholic acid, taurocholic acid, chenodeoxycholic acid, carnosol, nicotinic acid, and nicotinic acid mononucleotide; *g_Parabacteroides* showed extremely significant correlation with 14 differential metabolites (^**^p < 0.01) including cholic acid, taurocholic acid, chenodeoxycholic acid, nicotinic acid, and nicotinic acid mononucleotide; *g_Alistipes* had extremely significant correlation with 13 differential metabolites (^**^p < 0.01) including carnosic acid, cholic acid, and chenodeoxycholic acid; *g_Odoribacter* had extremely significant correlation with 11 differential metabolites (^**^p < 0.01) including carnosic acid, cortisone, and chenodeoxycholic acid. Among the 21 differential metabolites, chenodeoxycholic acid was extremely significantly correlated with 10 gut microbiota (^**^p < 0.01) including p_Firmicutes, p_Bacteroidetes, p_Proteobacteria, *g_Lactobacillus, g_Bacteroides, g_Alistipes, g_Odoribacter, g_[Prevotella], g_Prevotella* and *g_Parabacteroides*; carnosic acid was extremely significantly correlated with 10 gut microbiota (^**^p < 0.01) including p_Firmicutes, p_Bacteroidetes, p_Proteobacteria, *g_Lactobacillus, g_Bacteroides, g_Alistipes, g_Odoribacter, g_[Prevotella], g_Prevotella* and *g_Parabacteroides*; cholic acid showed extremely significant correlation with 7 gut microbiota (^**^p < 0.01) including *g_Lactobacillus, g_Bacteroides, g_Alistipes, g_[Prevotella], g_Prevotella* and *g_Parabacteroides* and *g_Roseburia*. Overall, the correlation analysis suggested that metabolites and gut microbiota could affect each other.

## 4. Discussion

AA exposure causes organ toxicity, especially to the liver leading to liver damage (28). If the liver injury cannot be reversed, hepatic structure and function will be permanently destroyed/hindered. AST and ALT are widely distributed in various tissues, especially in the liver. Increased AST levels can be used as an indicator of mitochondrial damage, while high ALT levels suggest membrane damage (29). When the liver function or structure is damaged, these transaminases are released from the liver increasing their serum levels. Concurrently, in case of liver damage, bile circulation is blocked, ALP activity increases, the utilization of free fatty acids decreases, and the level of free fatty acids in the blood increases, ultimately increasing serum TG levels. Therefore, the variation in serum ALP and TG levels indirectly reflects the degree of liver damage (30). Our results showed that the serum levels of ALT, AST, ALP, and TG levels were significantly increased in AA-induced mice, indicating serious liver damage (Figure 2). Interestingly, oral administration of KFP (best effect in KFP-M group) relieved the AA-induced liver injury as indicated by reverted serum levels of ALT, AST, ALP, and TG (Figure 2). Meanwhile, liver histopathological observation also correlated with biochemical results, indicating the recovery of damaged hepatocytes (Figure 3). Meantime, the body weight of AA-induced mice also significantly improved after KFP intervention (Table 1). AA toxicity disrupted liver function, which lead to nutritional dysfunction, and ultimately weight loss in AM mice, however, KFP intervention alleviated liver damage and thereby slowly improved body weight loss.

AA toxicity not only damages the liver but also destroys the balance of the host's gut microbiota. In this study, AA-induced reduction of Shannon and Simpson indices demonstrated a decrease in the diversity of gut microbiota, which was reversed by KFP-M intervention. AA exposure disturbed gut microbiota at the phylum level, increasing the abundances of Firmicutes and Actinobacteria while decreasing Bacteroidetes and Proteobacteria (Figure 4E). An increased abundance of Firmicutes in AA-induced mice was shown to promote glucose metabolism disorder, which is consistent with our results. However, KFP or APS intervention did not alter these changes (Figure 4E). Firmicutes and Bacteroidetes are the common inhabitants of animal and human gastrointestinal tracts, accounting for ~90% of total gut bacteria (31). Many of the Firmicutes are harmful, while some are beneficial bacteria. Further microbiome analysis revealed that AA exposure significantly decreased the abundance of *Lactobacillus, Ruminococcus*, and *Bacteroides* but increased *Allobaculum* and *Oscillospira*. Meanwhile, KFP-M or APS treatment significantly increased the abundance of *Lactobacillus* and decreased the abundance of *Oscillospira*. *Lactobacillus*, with beneficial probiotic functions, is a vital inhabitant of the gut microbiota (32). *Lactobacillus* can regulate the host's gut microbiota, improve immunity, and reduce serum cholesterol, blood pressure, and antioxidant stress levels (33). Besides, *Lactobacillus* act as a natural antioxidant to ameliorate liver injury (34). Many members of *Lactobacillus* have prevention and treatment effects against hepatic diseases. For instance, a combination of *L. plantarum* and *L. acidophilus* was shown to prevent liver injury by modulating the gut microbiota and reducing mitochondrial damage (35). Some recent studies showed that polysaccharides promote the proliferation of *Lactobacillus in vitro and in vivo*. For instance, persimmon polysaccharides accelerated the proliferation of *Lactobacillus acidophilus NCFM* and *Lactobacillus acidophilus CICC 6075 in vitro*; longan polysaccharides significantly promoted the proliferation of *Lactobacillus casei, Lactobacillus acidophilus, Lactobacillus plantarum*, and *Enterococcus faecalis in vivo* (36, 37). In this study, KFP-M and APS intervention significantly increased the abundance of *Lactobacillus* (Figure 4G). Moreover, LEfSen analysis showed that *o_Lactobacillales, f_Lactobacillaceae*, and *g_Lactobacillus* were the biomarker species of KFP-M mice (Figure 4H), indicating that KFP promoted the abundance of *Lactobacillus*. AA exposure increased the abundance of *Allobaculum* (Figure 4G), which is one of the most sensitive intestinal tract genera that changes with host dietary habits. *Allobaculum* was shown to have strong positive correlations with the host's body weight, IL-1β, and TNF-α levels (38, 39). In humans, *Oscillospira* is often increased in the fecal microbiome in case of liver injury (40). *Oscillospira* was significantly enriched in the lung cancer group, indicating its strong association with liver damage (41). Notably, KFP-M intervention decreased the abundance of *Oscillospira* in AM mice (Figure 4G).

Variations in gut microbiota are usually accompanied by metabolite changes (42). Several studies indicate that gut microbiota can regulate liver injury (43). The gut-liver axis is a bidirectional anatomical and functional interaction between the intestinal tract and liver, primarily through portal circulation (44). Altered levels and compositions of serum metabolites can indicate the physiological and pathological status of subjects (45). Therefore, we examined the effects of KFP-M intervention on mice metabolites by untargeted metabolomics. KFP-M intervention alleviated AA toxicity in AA-induced mice. In total, 59 potential biomarkers and 13 metabolic pathways were altered in AA-induced mice serum (Figures 6, 7). AA toxicity is known to alter serum metabolites (46), causing related diseases, such as liver injury (47). Metabolomics results demonstrated that KFP-M intervention modified the levels of nicotinic acid, nicotinic acid mononucleotide, niacinamide, D-glutamine, chenodeoxycholic acid, taurocholic acid, cholic acid, and L-aspartic acid in AM mice (Figure 6). Nicotinic acid, belonging to the vitamin B family, is formed from tryptophan and mainly participate in lipid metabolism, oxidation, and anaerobic decomposition. After intestinal absorption, nicotinic acid is converted into niacinamide by the amidation of niacin, which is closely related to many metabolic processes, including glucose glycolysis and pyruvate metabolism (48). KFP-M intervention reverted the normal level of nicotinamide in AA-induced mice. Moreover, the levels of nicotinic acid and nicotinic acid mononucleotide involved in nicotinate and nicotinamide metabolism were also reversed by KFP-M treatment (Supplementary Table S1). Chenodeoxycholic, taurocholic, and cholic acids are effective components of bile acids (BAs). In the liver, cholesterol is converted to 7α-hydroxycholesterol by CYP7A1, which is then converted to chenodeoxycholic, taurocholic, and cholic acids by sterol 12α-hydroxylase and CYP27A1 (49). BAs are closely related to enterohepatic circulation and their serum levels indicate the status of BAs metabolism (50). About 95% of BAs are secreted into the small intestine through the gallbladder and then reabsorbed into the liver through the enterohepatic circulation (51, 52). Therefore, the serum levels of BAs are direct indicators of their absorption in the intestine and uptake by the hepatic portal vein to the liver. AA toxicity was shown to alter BAs levels in rat liver and feces (53). Accordingly, we examined the effect of AA on BAs metabolism by serum metabolomics, which showed increased levels of chenodeoxycholic, taurocholic, and cholic acids in AM mice, while the levels of taurocholic and cholic acids decreased after KFP-M intervention (Figure 6). The significantly increased levels of chenodeoxycholic, taurocholic, and cholic acids in AM mice, indicated altered pathophysiology and metabolic disorder of the liver (54). The correlation analysis showed that chenodeoxycholic, taurocholic, and cholic acids were significantly correlated with p_Proteobacteria, *g_Lactobacillus, g_Alistipes, g_Parabacteroides* and *g_Roseburia* (Figure 8). Chenodeoxycholic and cholic acid were significantly correlated with p__Firmicutes, *g_Bacteroides, g_Odoribacter, g_[Prevotella]*, and *g_Prevotella*. A few intestinal isolates of *Lactobacillus* and *Bacteroides* were reported to participate in BAs esterification (55). The increased levels of chenodeoxycholic, taurocholic, and cholic acids in AM mice could be due to the decrease of *Lactobacillus* and *Bacteroides*. Polysaccharides can restore BAs metabolism. Rat administered with lotus seed-resistant starch showed increased hydrolysis of sodium taurocholate and reduced conversion of sodium taurodeoxycholate by increasing the abundance of *Bifidobacterium* and *Lactobacillus* in fecal microbiota (56). Similar findings were present in our study.

In conclusion, our results demonstrated the body weight, liver histopathology, and biochemical parameters were significantly restored by KFP intervention in AA-induced mice. Furthermore, microbiome and serum metabolomics analyses confirmed the hepatoprotective effect of KFP, which potentially worked *via* the gut-liver axis. A medium dose of KFP supplementation improved the health of gut microbiota and liver in AA-induced mice by altering the metabolism of BAs. Additionally, Spearman correlation analysis indicated that alterations in gut microbiota were highly relevant to key changes in metabolites. This study uncovers the hepatoprotective mechanism of KFP from a microbiome-metabolome perspective and strengthens its status as a functional food.

## Data availability statement

The original contributions presented in the study are included in the article/Supplementary material, further inquiries can be directed to the corresponding authors.

## Ethics statement

The animal study was reviewed and approved by Medical Ethics Review Committee of Xi'an Medical College.

## Author contributions

MC conducted most of the experiments under the guidance of XC and YS. KW and LC helped in data analysis. NL, DZ, and WJ carried out article correction. PG and NL helped in discussions about pathology. All authors have made a substantial contribution to the work and approved it.
