# Supplementary material for: Effects of kiwi fruit (Actinidia chinensis) polysaccharides on metabolites and gut microbiota of acrylamide-induced mice
Source: Front Nutr. 2023 Feb 6;10:1080825. doi: 10.3389/fnut.2023.1080825 (PMC9939636; doi:10.3389/fnut.2023.1080825)
Supplement: Supplementary file 1 [file Table_1.doc]

Table S1 Pathway enrichment and corresponding metabolites markers between AM versus CK groups, and KFP-M versus AM groups.

| Metabolic pathway | AM versus CK | | KFP-M versus AM | |
| --- | --- | --- | --- | --- |
|  | Increased | Decreased | Increased | Decreased |
| Nicotinate and nicotinamide metabolism | Nicotinic acid mononucleotide*; Niacinamide**; 1-Methylnicotinamide**; Nicotinic acid** | N.D. | N.D. | Nicotinic acid mononucleotide*; Niacinamide**; 1-Methylnicotinamide***; Nicotinic acid** |
| Primary bile acid biosynthesis | Chenodeoxycholic acid**；Allocholic acid**；Cholic acid** | Glycine***; Taurine*** | N.D. | Chenodeoxycholic acid**; Cholic acid**; Taurocholic acid* |
| Taurine and hypotaurine metabolism | Taurocholic acid* | Taurine*** | N.D. | Taurocholic acid* |
| Pyrimidine metabolism | N.D. | Uridine*; dUMP***; Deoxyuridine**; Uracil*** | Deoxyuridine**; Uracil*** | Deoxycytidine*** |
| Biosynthesis of unsaturated fatty acids | Eicosadienoic acid*; 8,11,14-Eicosatrienoic acid**; Gamma-Linolenic acid* | Erucic acid* | N.D. | Eicosadienoic acid***; 8,11,14-Eicosatrienoic acid***; Gamma-Linolenic acid**; Alpha-Linolenic acid* |
| Glycine, serine and threonine metabolism | N.D. | L-Serine*；Glycine***; Pyruvic acid*** | N.D. | Betaine** |
| beta-Alanine metabolism | N.D. | Hydroxypropionic acid***; Uracil*** | Uracil*** | N.D. |
| D-Arginine and D-ornithine metabolism | D-Ornithine** | N.D. | N.D. | D-Ornithine** |
| D-Glutamine and D-glutamate metabolism | D-Glutamine* | N.D. | N.D. | D-Glutamine*** |
| Arginine and proline metabolism | Citrulline*; L-Arginine** | 4-Acetamidobutanoic acid*** | 4-Acetamidobutanoic acid** | Citrulline* |
| Cysteine and methionine metabolism | N.D. | L-Serine*; Pyruvic acid*** | N.D. | L-Methionine* |
| Pantothenate and CoA biosynthesis | N.D. | Uracil*** | Uracil*** |  |
| Histidine metabolism | Urocanic acid* | N.D. | N.D. | Urocanic acid*** |
| Aminoacyl-tRNA biosynthesis | L-Arginine** | Glycine***; L-Serine* | N.D. | L-Aspartic acid***; L-Methionine* |
| Butanoate metabolism | N.D. | Pyruvic acid*** | N.D. | Succinic acid semialdehyde** |
| Alanine, aspartate and glutamate metabolism | N.D. | Pyruvic acid*** | N.D. | L-Aspartic acid***; Succinic acid semialdehyde** |
| Arachidonic acid metabolism | N.D. | 5-HETE*** | 5-HETE* |  |
| Steroid hormone biosynthesis | Cortisone** | N.D. | N.D. | Cortisone** |
| Lysine degradation | 4-Trimethylammoniobutanoic acid** | N.D. | N.D. | 4-Trimethylammoniobutanoic acid** |
| Tryptophan metabolism | L-Kynurenine* | N.D. | N.D. | L-Kynurenine* |
| Cyanoamino acid metabolism | N.D. | Glycine***; L-Serine* | N.D. | N.D. |
| Methane metabolism | N.D. | Glycine***; L-Serine* | N.D. | N.D. |
| Glutathione metabolism | N.D. | Glycine***; Pyroglutamic acid* | N.D. | N.D. |
| Valine, leucine and isoleucine biosynthesis | N.D. | Pyruvic acid*** | N.D. | N.D. |
| Pentose and glucuronate interconversions | N.D. | D-Xylitol* | N.D. | N.D. |
| Citrate cycle (TCA cycle) | N.D. | Pyruvic acid*** | N.D. | N.D. |
| Sphingolipid metabolism | N.D. | L-Serine* | N.D. | N.D. |
| Pyruvate metabolism | N.D. | Pyruvic acid*** | N.D. | N.D. |
| Glycolysis or Gluconeogenesis | N.D. | Pyruvic acid*** | N.D. | N.D. |
| Thiamine metabolism | N.D. | Thiamine* | N.D. | N.D. |
| Limonene and pinene degradation | N.D. | Perillic acid* | N.D. | N.D. |
| Nitrogen metabolism | N.D. | Glycine*** | N.D. | N.D. |
| Propanoate metabolism | Hydroxypropionic acid*** | N.D. | N.D. | N.D. |
| Fructose and mannose metabolism | L-Fucose* | N.D. | N.D. | N.D. |
| Porphyrin and chlorophyll metabolism | N.D. | Glycine*** | N.D. | N.D. |
| Purine metabolism | Xanthosine* | Adenosine* | N.D. | N.D. |
| Amino sugar and nucleotide sugar metabolism | L-Fucose* | N.D. | N.D. | N.D. |
| Valine, leucine and isoleucine degradation | L-Leucine*** | N.D. | N.D. | N.D. |
| alpha-Linolenic acid metabolism | N.D. | N.D. | N.D. | Alpha-Linolenic acid* |

Significant difference between groups was represented as ****p* < 0.001, ***p* < 0.01 and **p* < 0.05. N.D., no detected.
